# Effect of Glass Composition on Luminescence and Structure of CsPbBr_3_ Quantum Dots in an Amorphous Matrix

**DOI:** 10.3390/ma15051678

**Published:** 2022-02-23

**Authors:** Ruilin Zheng, Jumpei Ueda, Kenji Shinozaki, Setsuhisa Tanabe

**Affiliations:** 1 Graduate School of Human and Environmental Studies, Kyoto University, Kyoto 606-8501, Japan; ueda.jumpei.5r@kyoto-u.ac.jp (J.U.); tanabe.setsuhisa.4v@kyoto-u.ac.jp (S.T.); 2 National Institute of Advanced Industrial Science and Technology (AIST), Osaka 563-8577, Japan; k-shinozaki@aist.go.jp

**Keywords:** halide perovskites, CsPbBr_3_, structural evolution, glass matrix

## Abstract

Glass matrix embedding is an efficient way to improve the chemical and thermal stability of the halide perovskite QDs. However, CsPbX_3_ QDs exhibit distinct optical properties in different glass matrixes, including photoluminescence (PL) peak position, PL peak width, and optical band gap. In this work, the temperature-dependent PL spectra, absorption spectra, high-energy X-ray structure factor *S*(*Q*), and pair distribution function (PDF) were integrated to analyze the structural evolution of CsPbBr_3_ QDs in different glass matrixes. The results show that the lattice parameters and atomic spacing of CsPbBr_3_ QDs are affected by the glass composition in which they are embedded. The most possibility can be attributed to the thermal expansion mismatch between CsPbBr_3_ QDs and the glass matrix. The results may provide a new way to understand the effect of the glass composition on the optical properties of CsPbBr_3_ QDs in a glass matrix.

## 1. Introduction

Owing to excellent optical properties [1,2,3], all inorganic halide perovskites CsPbX_3_ (X = Cl, Br, I) quantum dots (QDs) have attracted great attention in various fields [4,5,6,7,8,9], especially in light-emitting diode (LED) lighting, lasers, and solar cells. However, due to the soft ionic nature of the halide perovskite QDs, improving their stability has always been an important research direction in practical applications [10,11,12,13]. The glass matrix embedding is an efficient way to improve the chemical and thermal stability of the halide perovskite QDs, which has attracted extensive research in recent years [14,15,16]. In addition to the traditional lighting application, CsPbX_3_ QDs in a glass matrix are also being developed for new applications such as three-dimensional (3D) laser printing and optical storage [17,18,19,20].

Notably, CsPbX_3_ QDs exhibit distinct optical properties in different glass matrixes, including photoluminescence (PL) peak position, PL peak width, PL lifetime, and optical band gap. The current mainstream view about distinct optical properties is that the glass compositions have an important effect on the cationic doping, interface modification, and crystallization kinetics change of CsPbX_3_ QDs in a glass matrix. For example, cationic doping of halide perovskite QDs can be achieved by adjusting the composition of the glass matrix [21,22,23,24], resulting in the observation of persistent luminescence from halide perovskite QDs [25]. The diffusion kinetics of precursor ions of CsPbX_3_ QDs can be changed by adjusting the glass network structure [26,27,28,29], which affects the optical properties of the QDs in a glass matrix. The heterogeneous pair-bonds are existing on the interface between the QDs and the glass matrix [30,31,32], which leads to the formation of defects on the QDs surface, thus affecting the optical properties of the QDs embedded in the glass matrix. In addition, the stability of CsPbX_3_ QDs in different glass matrixes is also distinct, which is believed to be determined by the resistance against hydration and oxygen of the glass matrix itself. For example, water-induced crystallization of halide perovskite QDs can be observed on the surface of fluorophosphate and phosphate glass with poor stability [33,34,35], while a similar phenomenon cannot be observed in the borosilicate glass matrix with higher chemical stability.

In general, the difference in the optical properties and stability of CsPbX_3_ QDs in different glass matrixes is attributed to the influence of external factors such as the interface with the glass matrix or the properties of the glass matrix itself. However, there were few reports about the structural evolution of CsPbX_3_ QDs in different glass matrixes. Due to the tiny size (5–10 nm) and low content of CsPbX_3_ QDs, it is difficult to accurately observe the structural and compositional changes of CsPbX_3_ QDs in a glass matrix by using X-ray diffraction (XRD) and X-ray photoelectron spectroscopy (XPS) techniques. Although CsPbX_3_ nanoparticles with large size can be obtained in the glass matrix by prolonging the heat treatment duration, thus improving the sensitivity of the signal detection for XRD and XPS. However, this method does not reflect the real structural evolution of the CsPbX_3_ QDs (5–10 nm) in a glass matrix. The high-energy X-ray total scattering technique has the characteristics of high sensitivity and resolution for structural evolution [36,37], thus monitoring the change of atomic distance in real space by calculating the pair distribution function (PDF). Additionally, in-situ temperature-dependent spectroscopy is another sensitive technique [38] to reflect the structural evolution by monitoring changes in optical properties of CsPbX_3_ QDs in a glass matrix.

In this contribution, we studied the structural evolution of CsPbBr_3_ QDs in the glass matrix by adjusting the types of glass network modifiers (alkaline earth metals) without significantly changing the B_2_O_3_-Al_2_O_3_-SiO_2_-based glass network structure. The temperature-dependent PL spectra, absorption spectra, high-energy X-ray structure factor *S*(*Q*), and PDF were integrated to monitor the structural evolution of CsPbBr_3_ QDs in the different glass matrixes. The results suggest that the lattice parameters and atomic spacing of CsPbBr_3_ QDs are affected by the change of the glass composition in which they are embedded. The most possibility can be attributed to the thermal expansion mismatch between CsPbBr_3_ QDs and the glass matrix. The results may provide a new way to understand the effect of the glass composition on the optical properties of CsPbBr_3_ QDs in a glass matrix.

## 2. Materials and Methods

The glass samples with the composition of 50B_2_O_3_-15SiO_2_-10Al_2_O_3_-15*R*F_2_-6PbBr_2_-4CsBr (*R* = Ca, Sr, Ba) were prepared by using a conventional melt-quenching method that employs covered platinum crucibles. All the raw materials (TCI, Tokyo, Japan), including B_2_O_3_, SiO_2_, Al_2_O_3_, CaF_2_, SrF_2_, BaF_2_, PbBr_2_, and CsBr, were mixed in stoichiometric proportions, then melted in an electrical furnace at 1050–1150 °C for 20 min at ambient atmosphere. The bubble-free glass melts were quenched on a cold stainless steel mold and pressed by another stainless steel plate to avoid crystallization. The obtained bulk glass samples were annealed at a muffle furnace near the glass transition temperature (*T*_g_) for 2 h, then cooled down to room temperature at a rate of 0.1 °C/min to remove the thermal strain. The as-prepared glass samples (G-Ca, G-Sr, and G-Ba) were cut and polished into small pieces with 2 mm thickness for optical measurements. The glass-ceramic samples (GC-Ca, GC-Sr, and GC-Ba) were prepared by subsequent heat treatment of the corresponding as-prepared glass samples (G-Ca, G-Sr, and G-Ba) for 20 h at 795 K. 

The low-temperature photoluminescent (PL) and absorption (Abs) spectra were measured by a CCD spectrometer (QE-65Pro, Ocean Optics, Orlando, FL, USA) with a 375 nm laser diode (LD) and a Xenon lamp (MAX-302, Asahi Spectra, Torrance, CA, USA) as the light sources, respectively. The temperatures of samples in the range of 20–300 K were controlled by a closed-circuit He cryostat (D105, Iwatani, Osaka, Japan). The absorption edge analysis was performed by using the Tauc plot method to distinguish the minor change of the absorption spectra at low temperatures. High-resolution X-ray scattering was measured at the BL08W beamline at SPring-8 (JASRI, Hyogo, Japan). The high-energy X-ray (115 keV) was irradiated to the glass-ceramic powders in a 2 mm diameter silica glass capillary, and the scattered X-rays were detected by a flat-panel area detector. The structure factor *S*(*Q*) and the pair distribution function (PDF) were calculated from the high-energy X-ray total scattering data [39].

## 3. Results

### 3.1. PL Spectra Evolution with Temperatures of CsPbBr_3_ QDs in the Glass Matrix

**Figure 1:**
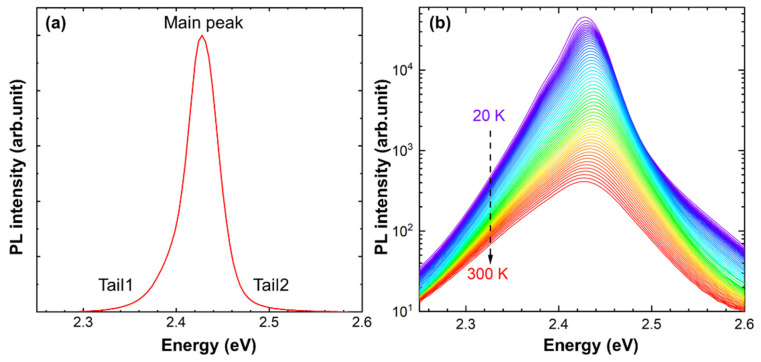
The PL spectra of GC-Ca (**a**) in a linear scale, (**b**) in a log scale at different temperatures.

Typically, there is one Gaussian peak and two tails in the PL spectrum of CsPbBr_3_ nanocrystals due to the exciton radiative recombination. As shown in Figure 1a, the PL spectrum of CsPbBr_3_ QDs in the glass matrix (GC-Ca) is similar to CsPbBr_3_ nanocrystals, which consist of the main peak and two weak tails at higher and lower energy sides, respectively. The slight spectral asymmetry in the high-energy side (Tail2) can be attributed to the self-absorption nature of CsPbBr_3_ QDs. The low-energy tail in the PL spectrum of semiconductors usually is related to the Urbach tail, which reflects the complementary information on the microstructure and defects. The temperature-dependent PL spectra in a log scale of GC-Ca were measured to analyze the Tail1. As shown in Figure 1b, the low energy tail in the PL spectra (in a log scale) of GC-Ca is nearly mono-exponential in the range of 20–300 K, which suggests that the Tail1 of CsPbBr_3_ QDs in the glass matrix is weak related to the Urbach Tail [40]. Compared with tails on both sides of the spectrum (Figure 1a), the main peak plays a dominant role in the change of spectral shape and PL peak position. Therefore, the influence of tails can be ignored in the evolution with temperatures of PL peak position and the full width at half maximum (FWHM).

**Figure 2:**
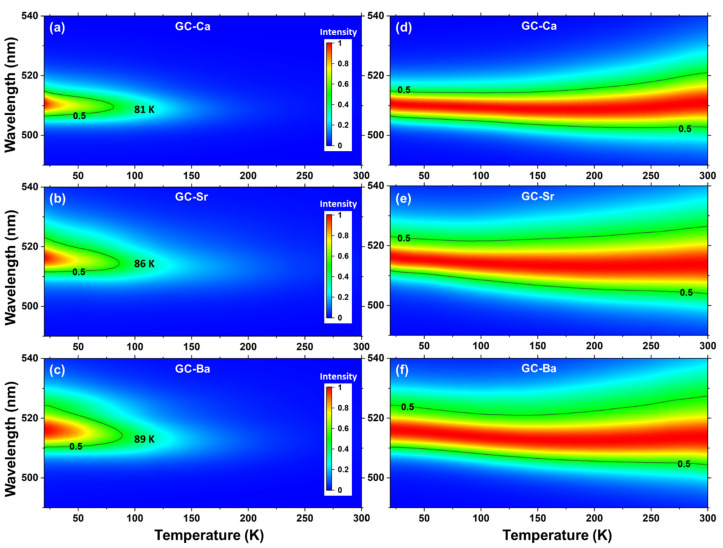
The PL intensity 2D mapping of (**a**) GC-Ca, (**b**) GC-Sr, and (**c**) GC-Ba with wavelength versus temperature. The normalized PL intensity 2D mapping of (**d**) GC-Ca, (**e**) GC-Sr, and (**f**) GC-Ba with wavelength versus temperature, setting the PL intensity of peak at different temperatures as the maximum value.

Figure 2a–c show the PL intensity 2D mappings of GC-Ca, GC-Sr, and GC-Ba, respectively. It can be observed that both the PL shape and intensity quenching trend of CsPbBr_3_ QDs in different glass matrixes present distinct evolution rules. As the radius of the alkaline earth ion increases from Ca to Sr and Ba in the glass matrix, the PL quenching temperature (50% PL intensity) of CsPbBr_3_ QDs increases from 81 to 89 K, while the width of the PL peak increases gradually. The normalized PL intensity 2D mappings (setting the peak at different temperatures as the maximum value) was performed to further analyze the PL peak position and width evolutions in the temperature range of 20–300 K. As shown in Figure 2d–f, the PL peak positions of CsPbBr_3_ QDs in different glass matrixes show significant redshift from 511 nm (GC-Ca) to 516 nm (GC-Sr and GC-Ba) at 20 K. With the increase of temperature, the PL peak positions of the GC-Ca present a slight blueshift in the range of 20–130 K, then turn to the redshift trend. In contrast, the PL blueshift trends of the GC-Sr and GC-Ba are more significant than GC-Ca at low temperatures, while the inflection point of the redshift increases. 

**Figure 3:**
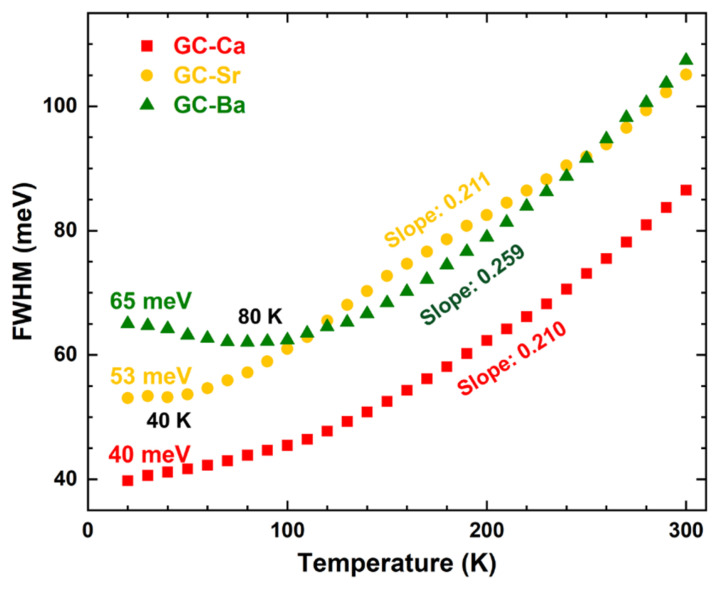
FWHM PL intensity of the GC-Ca, GC-Sr, and GC-Ba as a function of temperature.

Besides the low-energy tail, the FWHM of the PL peak also depends on the degree and nature of the disorder. Therefore, the FWHM were further used to analyze the structural evolution with temperatures of CsPbBr_3_ QDs in the glass matrix. The temperature dependence of FWHM PL intensity of the GC-Ca, GC-Sr, and GC-Ba are shown in Figure 3. The FWHM values at 20 K of CsPbBr_3_ QDs in the glass matrix are gradually increased from 40 meV (GC-Ca) to 53 meV (GC-Sr) and 65 meV (GC-Ba) with the glass composition change. Additionally, the FWHM of the GC-Ca monotonically increases with the increase of temperature. In contrast, the FWHM of the GC-Sr and GC-Ba show nonlinear change trends, which have minimum values of the FWHM at 40 and 80 K, respectively. When the temperature is higher than the inflection point of the FWHM evolution trend (no minimum value for the GC-Ca), the FWHM values of all the samples increase linearly. It is worth noting that the slope of the GC-Ba (0.259) is significantly greater than the GC-Ca (0.210) and GC-Sr (0.211), which suggests that the FWHM broadening rate of CsPbBr_3_ QDs in the GC-Ba is higher than that in the GC-Ca and GC-Sr during the heating process. 

### 3.2. Absorption Edge Evolution with Temperatures of CsPbBr_3_ QDs in the Glass Matrix

The temperature-dependence of absorption spectra was measured to analyze the optical band gap evolution at the temperature range of 20–300 K. The intensity values of (*αhν*)^2^ were calculated from absorption spectra by using the Tauc plot method to distinguish the minor change of the direct band gap at low temperatures. According to Tauc [41], the band gap energy can be obtained from the Equation (1):

(*αhυ*)^1/*m*^ = *B*(*hυ* − *E*_g_), (1)

where *h* is Planck’s constant, *υ* = frequency (s^−1^), *B* is a comparative constant, *E*_g_ = energy band gap (eV). *m* indicates the type of electronic with different values for direct (m = ^1^/_2_), direct forbidden (m = ^3^/_2_), indirect (m = 2), and indirect forbidden (m = 3), respectively.

**Figure 4:**
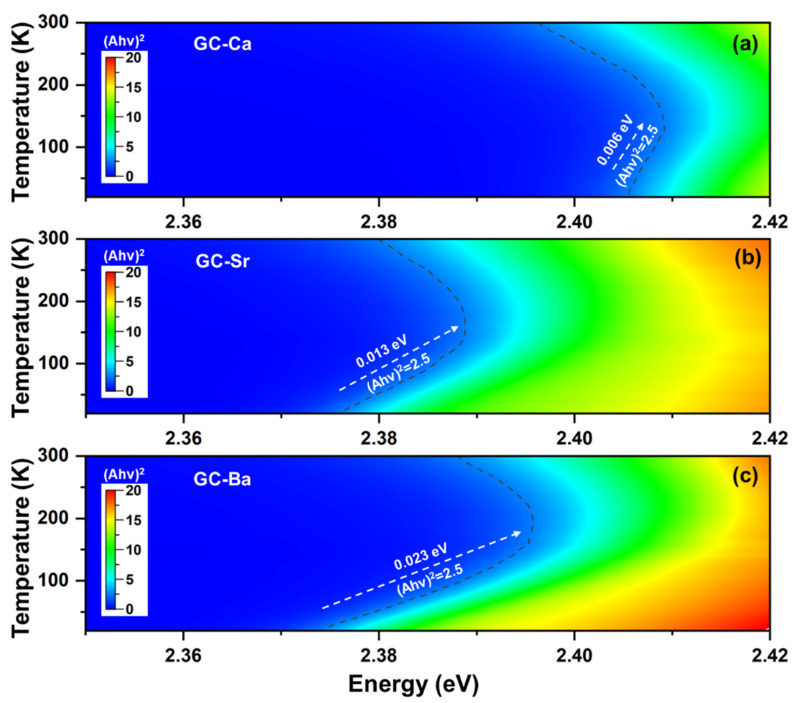
Temperature-dependent absorption edge evolution of (**a**) GC-Ca, (**b**) GC-Sr, and (**c**) GC-Ba in the heating process from 20 to 300 K at a temperature ramping rate of 5 K/min. The intensity values of (*αhν*)^2^ were calculated from absorption spectra by using the Tauc plot method.

As shown in Figure 4, the absorption edge of all the samples presents a nonlinear change, that is, the blueshift of the absorption edge turns to an abnormal redshift trend at high temperature after the inflection point. Additionally, the inflection point temperature of CsPbBr_3_ QDs also shows a significant change in different glass matrixes (120/140/160 K for GC-Ca/Sr/Ba). Because the direct band gap of CsPbBr_3_ QDs can be evaluated by the slope of absorption edge, we take the evolution of (*αhν*)^2^ value (2.5) as an example to analyze the change rules of absorption edge shift. As shown in Figure 4a, there is a 6 meV (Figure 4a) blueshift of the absorption edge occurring in the range of 20–130 K (GC-Ca), then turn to the redshift trend until the initial value is exceeded. As the Ca in the glass composition is replaced by Sr and Ba, the absorption edges of the GC-Sr and GC-Ba blueshift are 13 meV (Figure 4b) and 23 meV (Figure 4c), respectively. Furthermore, the energy shift of the GC-Sr and GC-Ba becomes weaker in the subsequent redshift process. All the results suggest that the optical properties of CsPbBr_3_ QDs are related to the glass composition in which they are embedded.

### 3.3. Crystal and Atomic Pair Structure of CsPbBr_3_ QDs in the Glass Matrix

**Figure 5:**
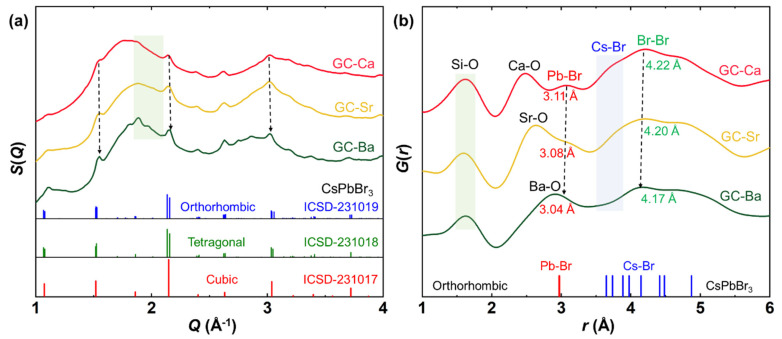
(**a**) High-resolution structure factor *S*(*Q*) and (**b**) the pair distribution function (PDF) curves *G*(*r*) of GC-Ca, GC-Sr, and GC-Ba at room temperature (where *Q* is the X-ray scattering vector).

High-energy X-ray scattering was used to analyze the crystal and the atomic pair structure of CsPbBr_3_ QDs in different glass matrices. The high-resolution *S*(*Q*) and PDF curves *G*(*r*) of the GC-Ca/Sr/Ba are shown in Figure 5. The fingerprint diffraction peaks of the perovskite CsPbBr_3_ can be found in the high-resolution *S*(*Q*) patterns of the GC-Ca/Sr/Ba (Figure 5a). Notably, the diffraction peaks of CsPbBr_3_ QDs show minor shape changes in different glass matrices, especially in the region of weak diffraction peaks (green region). Additionally, the diffraction peak width shows a narrowing trend as the alkaline earth in the glass composition is replaced from Ca to Sr and Ba, which means that the crystallinity of CsPbBr_3_ QDs is gradually increasing. In addition, the diffraction peaks of CsPbBr_3_ QDs slightly move to the high scattering vector as the atomic radius of alkaline earth increases in the glass matrix, which indicate that the unit cell shrink. 

The PDF was further used to analyze the pair information of the GC-Ca/Sr/Ba samples in the real space. As shown in Figure 5b, the *R*-O (*R* = Ca, Sr, Ba) pair distance gradually increases in the GC samples, which shows a good agreement with the atomic radius increasing trend of alkaline earth. In a contrast, the distance of the Si-O pair remains unchanged in all the samples. Therefore, we can conclude that the network structure of the glass matrix remains unchanged, and the only variable is the radius of the network modifier. Notably, the Pb-Br and Cs-Br pairs of CsPbBr_3_ QDs can be observed in the *G*(*r*) curves, which show a slight reduction in the distance with the increase of atomic radius (Ca, Sr, Ba). However, both the Pb-Br (3.04) and Br-Br (4.17) pairs of CsPbBr_3_ QDs in a glass matrix have a longer distance than in a single crystal. Therefore, we can conclude that the glass composition has a significant effect on the structure of CsPbBr_3_ QDs in a glass matrix.

## 4. Discussion

Generally, it is believed that the optical properties of the halide perovskite QDs can be adjusted by changing the composition of the glass matrix in which they are embedded, passivating the surface defects of the QDs or doping the cations in the perovskite structure. When the optical properties of the halide perovskite QDs are regulated by surface modification, the lattice structure will not change. In this case, the changes in the optical properties of the QDs are mainly reflected in PL lifetime and quantum efficiency. Other optical properties determined by the crystal structure, such as optical band gap and PL peak position, will not change significantly. In this work, the evolution of temperature-dependent absorption edge and PL spectra of CsPbBr_3_ QDs in different matrices are consistent with the change of lattice structure. Especially, the trends of absorption edge shift (Figure 4) and the FWHM (Figure 3) broadening were intensified with the increase of alkaline earth ion radius, which indicates that the change of optical properties of QDs is not only caused by surface modification. 

The PL of CsPbBr_3_ QDs originates from the exciton recombination process. The PL peak position and optical band gap evolutions with temperatures are related to lattice thermal expansion and phase transition [42]. Generally, PL peak position and band gap are linearly blue-shifted (widened) with the increase of temperature. When the phase transition occurs, the evolution trend of PL peak position and band gap will change due to the mutation of lattice parameters. In this work, both PL peak position and band gap evolutions are distinct when CsPbBr_3_ QDs are dispersed in different glass matrixes. Additionally, the PL spectra of the monodispersed halide perovskite QDs usually show a continuous broadening behavior with increasing temperature. This phenomenon was attributed to the important contribution of phonon scattering. According to this widely accepted mechanism, CsPbBr_3_ QDs should follow the same continuous broadening behavior (the trend and slope of FWHM) in different chemical environments. However, we found that not only the trend but also the slope of FWHM evolution (CsPbBr_3_ QDs) presents distinct behavior in different glass matrixes. For example, CsPbBr_3_ QDs show a monotonically increasing trend in the G-Ca matrix, while there are different minima in G-Sr (40 K) and G-Ba (80 K). 

The CsPbBr_3_ QDs embedded in a glass matrix perfectly realize the isolation of external environment factors. If the optical properties change of the GC-Ca/Sr/Ba is related to the internal cationic doping, the lattice parameter of CsPbBr_3_ QDs should follow the radius difference between the doping ions and Pb ions. For example, the Ba^2+^ doping will lead to the unit cell expanding, while the Ca^2+^ doping will lead to the unit cell shrink. Synchrotron X-ray total scattering data not only contain the structural diffraction signal of CsPbBr_3_ QDs but also atomic scattering information of the glass matrix. Therefore, we can analyze the structure of CsPbBr_3_ QDs and the glass matrix using *S*(*Q*) and PDF, respectively. As shown in Figure 5, the unit cell of CsPbBr_3_ QDs gradually shrinks as the radius of alkaline earth ions increases. Additionally, the distance of both Pb-Br and Br-Br pairs in CsPbBr_3_ QDs is decreasing in the GC-Ca/Sr/Ba matrixes, which means that lattice parameter change rules of CsPbBr_3_ QDs are opposite to the radius difference between the alkaline earth and Pb ions. The results rule out the possibility that the structural evolution of CsPbBr_3_ QDs is caused by the substitution of alkaline earth metals for Pb^2+^. 

According to the evolution of both optical properties and structural characteristics, we can conclude that there is another external factor affecting the structure of CsPbBr_3_ QDs in a glass matrix. As the only variable in different glass matrixes is the alkali earth element radius, the thermal expansion coefficient of the glass matrix increases gradually with the increase of the element radius. In this case, the thermal expansion mismatch between CsPbBr_3_ QDs and the glass matrix decrease as the radius of alkaline earth ions increase. Hence, the volume of CsPbBr_3_ QDs in a glass matrix decrease with the decrease of thermal expansion mismatch, and all of them (Figure 5) are larger than monodispersed nanoparticles. Unlike monodispersed QDs, the spontaneous thermal expansion of QDs in a glass matrix is affected (suppressed/reinforced) by the mismatch between the matrix and QDs. Therefore, we suggested that the difference of thermal expansion mismatch value leads to the distinct optical properties of CsPbBr_3_ QDs in different matrixes [40]. Additionally, the abnormal PL linewidth evolution at low temperatures (120–160 K) can also be attributed to the change of phase transition temperature caused by thermal expansion mismatch.

In this work, we analyzed the effect of local strain on PL and structure evolutions by using a glass matrix embedding strategy. The thermal quenching process (Figure 2a–c) was usually attributed to the combination of thermally activated carrier trapping and phonon-assisted non-radiative decay. Besides the local strain, the possible defects of halide perovskite QDs in a glass matrix are also related to structural disorders such as vacancies, domains formation, and lattice distortion [43]. The suppression of temperature quenching and improvement of thermal stability can be achieved using the surface passivation strategy for monodispersed halide perovskite QDs [44]. The surface defects of the QDs in a glass matrix are determined by the glass network structure and the pair-bond of the interface. When the operating temperature is lower than the transition temperature (*T_g_*) of the glass matrix, the glass network structure and pair-bond of the interface remain stable during the heating process. Therefore, we are not yet able to determine the specific defect category of halide perovskite QDs in the glass matrix. We will continue to explore the detailed mechanism of optical and structural evolutions of halide perovskite QDs in a glass matrix in our future work.

## 5. Conclusions

In summary, we explored the optical and structural evolutions of CsPbBr_3_ QDs in the glass matrix. By adjusting the types of glass network modifiers (alkaline earth metals), the B_2_O_3_-Al_2_O_3_-SiO_2_-based glass network structure remains. The temperature-dependent PL spectra, absorption spectra, high-energy X-ray structure factor *S*(*Q*), and PDF were integrated to monitor the structural evolution of CsPbBr_3_ QDs in the different glass matrixes. The results ruled out that the distinct optical and structural evolutions of CsPbBr_3_ QDs in different glass matrixes are determined by the ions doping in which they are embedded. The most possibility of the abnormal evolutions can be attributed to the thermal expansion mismatch between CsPbBr_3_ QDs and the glass matrix. The results may provide a new way to understand the effect of the glass composition on the optical properties of CsPbBr_3_ QDs in a glass matrix. 

## Data Availability

The data presented in this study are available on request from the corresponding author.
